# Salvage of undeflatable intra-stent angioplasty-balloon-catheter with direct percutaneous needle puncture

**DOI:** 10.1186/s42155-025-00638-8

**Published:** 2025-12-31

**Authors:** Leonardo Pasquetti, Edoardo Pasqui, Giuseppe Galzerano, Elisa Lazzeri, Bruno Gargiulo, Gianmarco de Donato

**Affiliations:** Vascular Surgery, Department of Medicine, Surgery and Neuroscience, University of Siena, Viale Bracci, Siena, Italy

**Keywords:** Undeflatable balloon, Direct puncture, Stent, CTO, SFA

## Main text

### Background

To the Editor,

Balloon angioplasty and stenting are standard components of endovascular treatment for peripheral arterial disease. Although balloon inflation and deflation are routine steps, failure of a balloon to deflate is an exceptionally rare but potentially dangerous complication [1]. Evidence from coronary interventions exists, but applicability to peripheral vessels is limited due to differences in vessel calibre, calcium burden, and device design [2]. Here, we describe a case of balloon deflation failure inside a stented superficial femoral artery (SFA), successfully managed with a direct percutaneous puncture.

### Case presentation

**Fig. 1:**
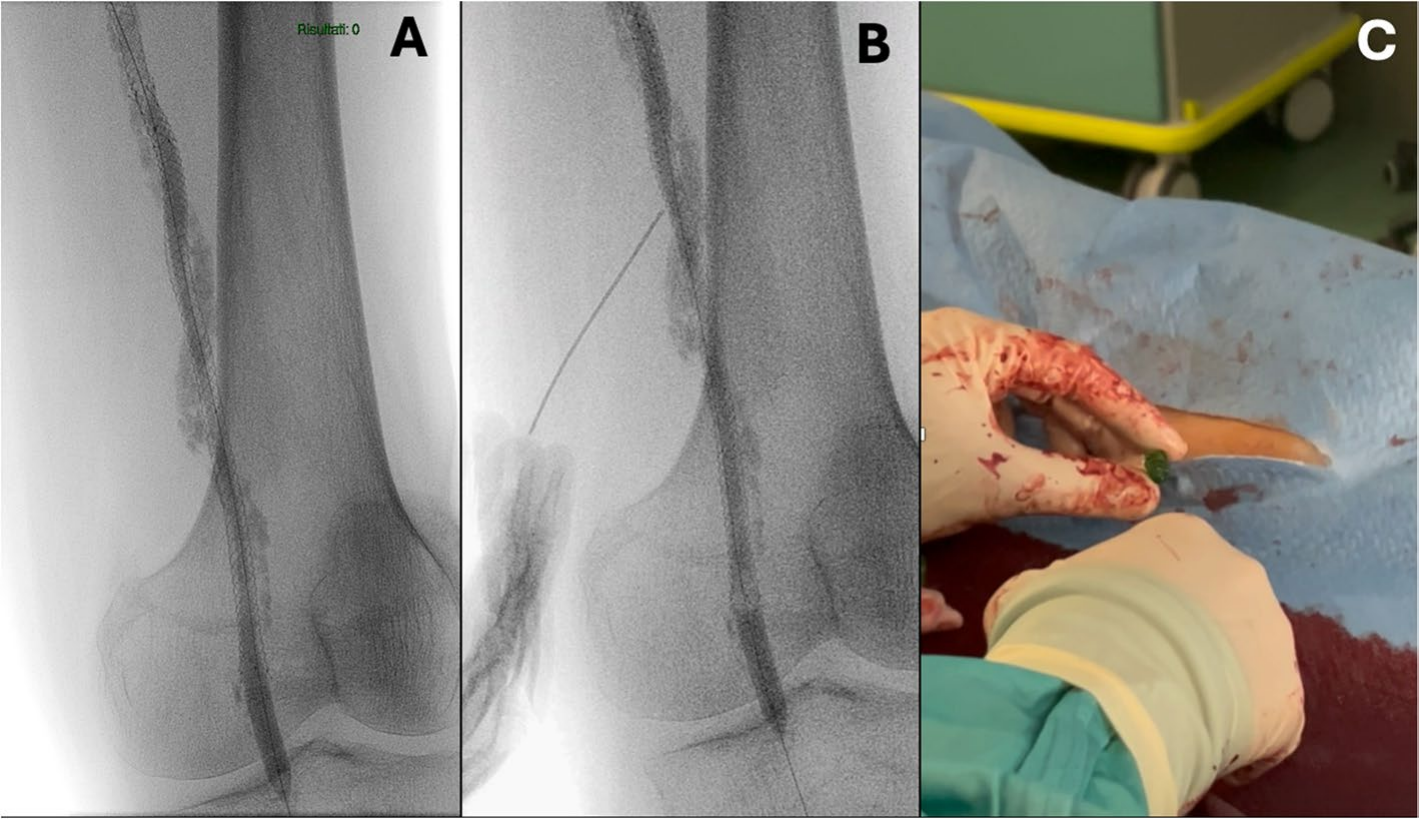
During stents post-dilation, upon completion of the inflation phase, the balloon failed to deflate despite repeated attempts using standard techniques. **A** The balloon catheter results in being undeflatable. **B** Direct puncture of the balloon catheter through the stent’s struts is performed under X-ray guidance. **C**: back “bleeding” (saline and contrast medium) is observed. Aspiration through the needle successfully facilitated the deflation of the balloon catheter

**Fig. 2:**
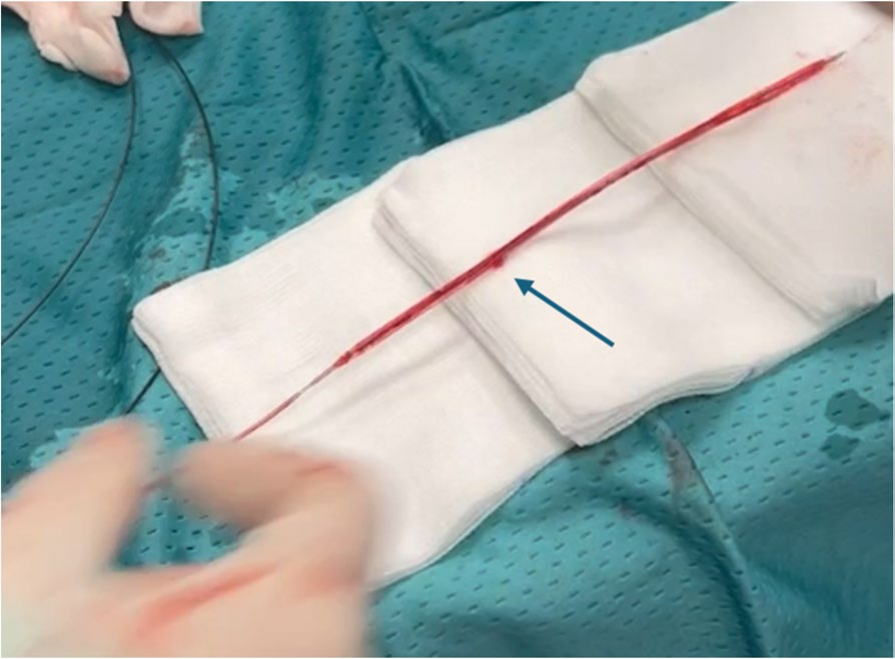
At the end of the procedure, by inflating the balloon catheter, the site of the percutaneous puncture is observed. The rest of the device appeared intact

A 65-year-old man with diabetes, dyslipidaemia, heavy smoking history, and chronic limb-threating ischemia (Rutherford V) presented with recurrent ulceration of the left forefoot stump. Angiography showed chronic total occlusion (CTO) of a previously stented SFA. The lesion was crossed and prepared with plain-old balloons (Advance Serenity 5 × 150 mm and 6 × 150 mm, Cook Medical, Bloomington, IN, USA) and a non-compliant balloon (Dorado 7 × 150 mm, BD, Franklin Lakes, NJ, USA) and then relined with two 6 × 170 mm Pulsar-18 T3 stents (Biotronik SE&Co. KG, Berlin, Germany). Post-dilation was performed with the previously used 6-mm hydrophilic-coated over-the-wire balloon. At the end of inflation, the balloon failed to deflate despite repeated attempts with standard measures, including repeated flushing and inflation above the rated burst pressure. The fully expanded balloon obstructed blood flow and could not be withdrawn without risking stent deformation or vascular injury (Fig. 1A). A percutaneous bailout technique was adopted. Under fluoroscopic guidance in a single-plane projection, a 21-gauge, 7-cm microset puncture needle with a transitionless-tip design was advanced through the mid-thigh skin and vessel wall to directly puncture the balloon through the stent struts (Fig. 1B). Subsequently, back “bleeding” (the mix of saline and contrast medium) was observed (Fig. 1C). Passive deflation was insufficient, so aspiration with a 10-cc syringe enabled complete balloon collapse. The catheter was removed intact. Reinflation outside the body confirmed the puncture site without other structural defects (Fig. 2). Final angiography demonstrated no arterial injury or recoil, with restored SFA patency.

## Discussion

Entrapment or inability to deflate a balloon is exceedingly uncommon, especially in peripheral interventions. In the coronary field, multiple bailout techniques have been described: intentional rupture via overinflation, high tip-load guidewire puncture, cutting the hypotube to restore luminal continuity [3], guide extension-mediated puncture [4], buddy balloon displacement [5], and excimer-laser perforation [6]. In our case, conventional measures were ineffective. Therefore, we opted for the direct puncture technique. The most plausible mechanism of deflation failure was hypotube damage, which prevented pressure transmission to the balloon. Such damage may occur during retrieval and reinsertion or when advancing through stent struts, heavily calcified CTO [7], or tortuous vessels. The presence of stents likely increased the risk of kinking or microperforation of the shaft. This case highlights the importance of careful balloon manipulation, particularly in previously stented vessels, and the need to replace any device showing resistance or suspected structural damage. When balloon deflation failure does occur, direct puncture represents, in percutaneously accessible vessels, a simple, rapid, and reproducible bailout technique.

## Data Availability

Data sharing is not applicable to this article as no datasets were generated or analyzed during the current study.

## Abbreviations

CTO: Chronic total occlusion
SFA: Superficial femoral artery

## References

[1] Haidar HA, Perier M, Benamer H. How to manage an entrapped undeflatable coronary balloon. Ann Cardiol Angeiol (Paris). 2024;73(4):101779. 10.1016/j.ancard.2024.101779. Epub 2024 Jul 23. PMID: 39047394. 39047394 10.1016/j.ancard.2024.101779

[2] Guedeney P, Claessen BE, Mehran R, Mintz GS, Liu M, Sorrentino S, et al. Coronary calcification and long-term outcomes according to drug-eluting stent generation. JACC Cardiovascular interventions. 2020;13(12):1417–28. 10.1016/j.jcin.2020.03.053. 32553329 10.1016/j.jcin.2020.03.053

[3] Watt J, Khurana A, Ahmed JM, Purcell IF. Simple solution for an undeflatable stent balloon in the left main stem. JACC Cardiovasc Interv. 2015;8(14):e245-6. 10.1016/j.jcin.2015.07.033. Epub 2015 Nov 18. PMID: 26604055. 26604055 10.1016/j.jcin.2015.07.033

[4] Takama T, Ito Y, Ishimori H, et al. Failure of a balloon to deflate during post dilatation in a coronary artery. Cardiovasc Interv Ther. 2015;30:57–60. 10.1007/s12928-014-0249-5. 24532231 10.1007/s12928-014-0249-5

[5] Nosair M, Hayek A, Avram R. Get out of jail: managing an entrapped balloon using subintimal plaque modification. JACC Case Rep. 2022;4(19):1252–5. 10.1016/j.jaccas.2022.07.004. PMID: 36406910; PMCID: PMC9666741. 36406910 10.1016/j.jaccas.2022.07.004 PMC9666741

[6] Savvoulidis P, Bagur R, Ybarra LF. Retrieval of undeflatable stent balloon using laser energy. Cardiovasc Revasc Med. 2021;28:136–9. 10.1016/j.carrev.2020.10.024. Epub 2020 Nov 2. PMID: 33168432. 10.1016/j.carrev.2020.10.024 33168432

[7] Fereydooni A, Chandra V, George EL. Endovascular retrieval of an entrapped balloon in a tibial artery. J Vasc Surg Cases Innov Tech. 2024;10(3):101459. 10.1016/j.jvscit.2024.101459. PMID: 38591015; PMCID: PMC10999707. 38591015 10.1016/j.jvscit.2024.101459 PMC10999707

